# Quarterly Summary

**Published:** 1857-04

**Authors:** 


### QUARTERLY SUMMARY.

### ANATOMY AND PHYSIOLOGY.

1.—*Minute Anatomy of the Liver.*—Dr. Lionel S. Beale has published, (*Med. Times and Gaz.*, July 26, 1856,) several interesting lectures on this subject. The following is a summary of the most important points brought under notice:

"The livers of all vertebrate animals are penetrated in every part by two sets of channels, which alternate with each other. One series, *portal canals*, contains a branch of the portal vein, hepatic artery, and hepatic duct, *interlobular;* and the other series, *hepatic venous canals*, is occupied by a single branch of the hepatic vein, *intralobular*.

The vessels ramifying in the portal canals are ultimately distributed in such a manner that they serve to divide the organ into little masses, and thus map out spaces, or *lobules*, each of which contains all the structural elements of the organ, and be regarded as an elementary liver.

In the intervals between the fissures by which the portal vein, artery and duct are conducted to the lobule, its capillary vessels and its secreting structure are continuous with those of adjacent lobules.

The size and form of the lobules differ much in different animals; but their essential structure is the same in all, except in the pig, in the polar bear, according to Müller, and in the octodon cummingii, (one of the rodents,) according to Hyrtl.

In the pig each lobule is provided with a separate fibrous capsule of its own, and is, therefore, completely isolated from its neighbors. The portal vessels, artery and duct run between them, and give off branches to contiguous lobules. In the intervals between the fibrous capsules areolar tissue can frequently be demonstrated.

In all cases, upon a section, the lobule is seen to be bounded externally by branches of the vein, artery and duct, and in the center is situated a small branch of the hepatic vein.

In the liver of the human subject, and in that of vertebrate animals generally, with the exceptions above mentioned, the lobules are not separated from each other by any fibrous partition, and there is no areolar tissue or prolongation of Glisson's capsule between them, or in their interior.

The vessels at their entrance into the liver, and as they run for some distance in the larger portal canals, are surrounded with much areolar tissue; but the disposition of this texture about the vessels of the liver is very similar to its arrangement about the vessels distributed to other organs.

The *lobule* itself is composed of a solid capillary network, and of another network composed of a delicate tubular membrane, in which the liver cells are contained.

These networks mutually interwine with each other.

The capillary network is directly continuous with the smallest branches of the portal vein, distributed upon the circumference of the lobule on the one hand, and with the small intralobular vein arising in its centre upon the other. The vessels of the network converge towards the intralobular vein.

Small branches of the artery open into the venous capillaries of the lobule, near its circumference, and the diameter of these small branches is considerably less than that of the venous capillaries into which they open; the former not more than the 1-4000th of an inch in diameter, the latter about the 1-1600th.

In all cases, the blood enriched with constituents recently absorbed from the intestine, flows with a gradually increasing rapidity from the circumference of the lobule towards its centre, while the bile flows in a precisely opposite direction.

The cell containing network is directly continuous with the most minute ducts, which ramify at the circumference of the lobule, and it terminates in the centre by loops, which lie close to the intralobular vein.

The liver-cells lie within a tubular network of basement membrane, which separates them from the walls of the capillaries. In many cases, however, these thin membranes cannot be separated, and are, no doubt, incorporated with each other.

The cells are not attached to the basement membrane of the tube, but lie in its cavity. Among them free oil globules and granular matter are often found. Usually, there is only room for one row of cells, but sometimes two or more lie across the tube. In the embryo, in young animals, and in fishes, there is room for several rows to lie transversely across the tubes of the cell containing network.

The cells near the margin of the lobule take the most active part in the formation of bile. The secretion passes along the tubes in the slight interstices between the cells and the basement membrane, and colored fluid can be forced along these same interstices in a direction the opposite to that in which the bile flows during life, and, therefore, at a great disadvantage. The amount of space is in great measure determined by the quantity of blood in the vessels, and it is liable to great alteration.

The secreting tubes of the network are many times wider than the narrow thin-walled ducts with which they are directly continuous.

The smallest ducts are lined with a very delicate layer of epithelium, composed of flattened cells of a circular form, contrasting remarkably with the large secreting cells, which are not arranged in any definite manner within the tubes of the network.

The tubes of the cell containing network are about the 1-1000th of an inch in diameter, or more, but the finest ducts are not more than 1-3000th, and they are often seen even less.

The smallest ducts in some animals branch very freely, and the branches communicate with each other at intervals. In others they pursue a long course without branching, and in the pig they form an intimate network upon the surface of the lobule. In fatty livers of the pig, however, this ductal network often contains liver cells loaded with oil globules.

As the ducts increase in size they are provided with a fibrous coat, and the epithelium in their interior becomes columnar.

"The *interlobular ducts* do not anastomose.

When the fibrous coat reaches a certain degree of thickness, it contains numerous little cavities or *sacculi*, arranged entirely round the tube in the pig and in most animals, but forming two parallel rows, one on either side of the duct, in the human subject.

These little sacculi often communicate with each other in the coats of the duct. The smaller branches of the duct also anastomose frequently either in the coats of the duct or just external to them.

The sacculi appear to serve the purpose of bringing the bile in the thick walled ducts into closer relation with the vessels which surround them, and especially with the branches of the artery which are distributed to their coats.

In the transverse fissure of the human liver and some others, and in the large portal canals, are found some peculiar branches of the duct, *vasa aberrantia*, with numerous sacculi on their walls, which anastomose with each other and form a network.

In the same localities in the human subject, and in the gall-bladder, a very peculiar arrangement of the vessels occurs. Both arteries and veins form a network, and each branch of the artery is accompanied with two branches of the vein, one on either side of it.

The vertebrate liver is to be regarded as a true gland, its secreting structure consisting of a *formative portion* taking the form of a network, and of a system of very narrow *efferent ducts* directly continuous with it. The secreting cells lie within a delicate tubular network of basement membrane, through the thin walls of which they draw from the blood the materials of their secretion, and they are thus brought into closer relation with, and are more nearly surrounded by the blood than the cells of any other secreting gland.

2.—*The True Spinal Marrow the True Sympathetic.*—Dr. Marshall Hall maintains (*Lancet*, July 12, 1856) that the true spinal marrow is the true great sympathetic, or rather the great dienergetic nerve of the general system, whilst the ganglionic intra and extra ganglionic system are its branches.

"Since," he says, "the promulgation of the diastaltic nervous system, of which the true and real centre is the spinal marrow and the spinal marrow only, two mistakes have been committed; the first was, to ascribe a similar function to the cerebrum; the second, to ascribe a reflex power to the ganglia. The former error arose from confounding the effect of *emotion* with those of the excitants of diastaltic action; the latter was, I believe, a mistake without foundation of any kind.

If we see a disgusting object, we experience an emotion which may issue in sickness and vomiting, as other emotions issue in sickness and vomiting. But who does not instantly perceive the difference between this psychological fact and the vomiting induced by the physical excitement of the fauces, for example?

Of an instance of a reflex action through the medium of the ganglionic system, the spinal centre being intercepted, not a trace has been discovered.

Is there in effect, any fact of a physical direct or reflex action, excited from, or through the substance of the cerebrum, freed from its membranes; or of any part of the ganglionic system, the spinal centre being removed? I believe not. To speak, then, of reflex actions of the cerebral or of the ganglionic systems, is to confound things essentially distinct, and unwarrantably to extend the use of terms recently introduced and well defined.

The great experimental question is this: When the cerebral and the spinal centres are removed, is there any possibility of inducing any phenomena such as those which have for ages been denominated *sympathetic?* This question has never been proved or discussed fully and distinctly. It might be resolved in the following manner:

The spinal marrow may be partially and even entirely divided in reptiles, the low-feeding and low-breathing fishes, and very young animals. This being accomplished, every means of inducing effects on the remaining functions is to be tried. If such effects be produced, it must be through the ganglionic nerve; if such effects be impossible, it must be because the real centre of reflex action is absent.

I have destroyed the whole cerebrum and spinal marrow in frogs, carefully avoiding intra-spinal hemorrhage; but I could not afterwards influence the action of the heart, or the phenomena of the circulation, by any means I could devise.

Still I regard the whole experimental question as requiring to be subjected to new investigation.

I formerly regarded the diastaltic action as limited to obvious movements; but a multitude of facts show that it has a vastly more extended application.

We all know the intimate relation between the ovarium, the uterus, and the mammæ. If the new born infant be put to the nipple, contraction of the uterus is excited. No one doubts that this is a reflex or diastaltic spinal action.

Pregnancy induces enlargement of the mammæ, and excites the secretion of milk. Is this secretory action less *spinal* than the former one?

The same derangement of the stomach induces convulsion, cramp, asthma, irregularity of the heart's action, altered secretion of the kidneys. Are they not all and equally diastaltic and spinal actions?

The same pregnancy which affects the mammæ induces nausea and vomiting. The last phenomenon is indubitably diastaltic spinal. Are the others less so?

Coldness impressed on the skin induces contraction of the rectum, and of the bladder, augments the action of the kidneys, stays hemorrhage, and induces various internal inflammations. I know a patient in whom damp feet would induce sneezing instantly, with increased secretion of mucus.

The whole cutaneous surface is simultaneously contracted or relaxed by the local and partial application of cold or hot water. I knew one patient, a near relative, in whom exposure to damp infallibly produced renal hemorrhage. A similar cause is apt to induce diarrhea.

Many other facts of the same kind might be adduced.

That secretion *is* influenced by diastaltic action through the spinal centre is now placed beyond all doubt by the remarkable experiment of M. Bernard, in which the glycogenic function of the liver is proved to be a diastaltic spinal action. I quote M. Bernard's words:

"Le nerf (pneumogastrique) porte au centre cérebro-spinal les sensation (?) internes emanées de sa périphérie ; l'excitation qu'il transmet est, dans se cas, *centripète*, et non pas centrifuge. Et, non effet, après avoir coupé le pneumogastrique, si, au lieu d'agir sur le bout périphérique, ce qui n'a aucun effet sur la sécrétion du sucre, on excite avec le galvanisme l'extrémité qui se rend à la moëlle, la fonction glycogénique non seulement n'est pas interrompue dans le foie, mais elle peut même être exagérée lorsque l'excitation a été poussée assez loin." *

As it is my present object only to suggest the idea, and to add a suggestion and an observation or two, I conclude this brief communication with one final remark. It is not only obvious that the true spinal marrow is in reality the true sympathetic, but that the diastaltic system has an extension over the animal economy scarcely yet contemplated by the physiologist and the physician. It is in reality, in this latter respect, only second to the circulation of the blood itself. As the blood really describes a *circle*, the diastaltic spinal system describes a *cycloid;* as the blood diffuses its atoms into every minute space of the system, the diastaltic spinal system extends its influence over every

* Leçons de Physiologie, p. 325. Paris, 1855.

one of those atoms! The blood undergoes its changes in the methæmatous, or blood changing (or capillary) channels placed between the ultimate branches of the arteries and the incipient roots of the veins; to these same points the diastaltic spinal system extends its wondrous influence.

---

3.—*On the Antrum Pylori in Man.*—By Prof. A. Retzius.—Many writers on anatomy, in the description of the human stomach, comprehend, under the denomination "antrum pylori" (Pförtner Höhle, cul-de-sac pylorique,) a portion of the viscus adjoining the pylorus; many do not mention this part; and others allude to it in a very cursory manner. I had been long engaged in dissections of the human body, without paying any special attention to it. Subsequently, I found, in examining the stomach in animals considered to have a single stomach, that the pyloric portion constituted a perfectly distinct part, and that in most vertebrate animals it has a peculiar structure, different from that of the rest of the organ. Many years ago I investigated and described, in the *Transactions of the Royal Academy of Sciences* (K. Vet. Acad. Handl.) for 1839, the structure of the stomach in some herbivorous rodentia; subsequently, I have likewise in man, time after time, discovered a certain almost regular ampullæ in this region, as well as, in many cases, a well-defined division, which I had no reason to regard as a morbid formation. The denomination "antrum pylori" appeared to me to indicate that its origin was based on observations resembling my own, and I therefore endeavored to trace the source of the name. I then found that Cruveilhier (*Traité d'Anatomie descr.*, tom. iii. p. 281,) who took the same view of the subject as I did, attributes the term to Willis, as also that Haller (*Elementa Physiol.*, tom. vi. lib. xix. sect. i. §3, "Ventricula figura") quotes the work and place where Willis employs the name. For his own part, Haller says on this subject, in the section of his work referred to: "Non raro aliqua strictura quasi divisus [here he cites Morgagni, and brings forward several instances of stomachs divided by strictures] maxime posterius, tum paulo cis pylorum, unde tunc *antri* aliqua imago nascitur (Willis) quam aliqui clarisimi viri nimis fecerunt."

Willis' work, where the name "antrum pylori" occurs, and where it seems to have its origin, is his *Pharmaceutices rationalis sive dia-*

*tribœ midicamentorum operationibus in corpore humano*, &c., cap. ii. "Partium intra quas medicamenta operari incipiunt, descriptio, usus et affectiones." The most important part, in my opinion, touching the point in question, is that in which the author speaks of the destination of the pylorus, where it is said: "Pylori munus est, non tantum contenta affatim et simul in magna copia ad intestina transmittere (quod quidem in catharsi et diarrhœa frequenter facit,) sed potius chylum satis confectum, in *sinum* suum excipere, aliquamdiu continere, et dein paulatim et per minutas portiones excernere. Enim vero hujus *antrum longum et capax quidam in ventriculo recesses et diverticulum esse videter*, in quod massæ chylaceæ portio magis elaborata et perfecta secedere, et inibi manere queat, donec alia crudior, et nuperius ingesta in ventriculi fundo plus digeratur," &c. We see from this, as well as from many other passages in the same work, that Willis attached much importance to this division of the stomach.

On a superficial examination, the human stomach appears to be a very simply constructed conical sac, from the form of which anatomy seems not to have much to gain. When, however, we consider the elaborate functions this sac has to perform, both in man and animals, and the numerous divisions and remarkable forms it exhibits in a great number of the latter, with many circumstances, both in health and disease, difficult of explanation, we are soon led to the conviction that very ingenious arrangements must be disposed in this apparently simple structure.

So far as I can recollect, there is not one of the modern writers who has, according to my view, described the pyloric portion of the stomach better than Cruveilhier. After having spoken of the pylorus itself, he says: "It is in this neighborhood of this construction (pylorus,) at about the distance of an inch, that the stomach, bending strongly on itself, forms on the side of the great curvature a very decided elbow, *coude de l'estomac,* and presents an ampulla corresponding to an internal cavity, designated by Willis by the name of antrum of the pylorus, &c. It is not unusual to see a second ampulla beside the first, and a third, but smaller, at the side of the lesser curvature, in consequence of the bend described by the stomach. These ampullæ, scarcely appreciable in a great number of subjects before insufflation, become very distinct, and even, in some subjects, very considerable, by distension," &c.

According to my experience, this part occurs principally under three forms. The first is that sketched in the description just now quoted from Cruveilhier; the second, in which the part is more elongated, is mentioned by Willis, when he says, "Antrum longum et capax;" the third, which may be called the conical variety, is that in which both the ampullæ here referred to by Cruveilhier, and their boundaries, are little marked, while the part itself is more conical.

In the first or shorter form, the part of the pylorus at the base is nearly as broad from the lesser to the greater curvature, as it is long, has two ampullæ at the lesser curve, and most frequently one at the greater, anterior to the great flexure, or *coude de l'estomac.* The first ampulla in the lesser curve is bounded at the thicker end by a deep indentation, which exactly corresponds to the great flexure just mentioned, the *coude de l'estomac*, and at the narrower end by a shallower indentation, separating it from the second ampulla, lying next to the pylorus. The ampulla in the greater curve is separated from the *coude de l'estomac* by a shallow indentation, often amounting only to a depression extending half way round; this ampulla is usually somewhat larger than the corresponding one on the lesser curve, and, like it, reaches to the proper pylorus.

The entire of this part of the stomach is usually provided with a very thick muscular coat. It is particularly the circular layer of the latter which gives the pyloric portion its predominant thickness. The external longitudinal fibres lie here almost as on the colon, collected in bands, one on the anterior, and one on the posterior surface; these bands are not, however, as on the colon, distinctly bounded, but are only denser collections of bundles of muscular fibres, which, both anteriorly and posteriorly, become thinner, and dispersed over all the surrounding parts. This similarity to the tæniæ valsalvæ on the colon, first observed, as it seems, by Helvetius (*sur la digestion,*) gave rise to the now obsolete name, *ligamenta pylori.* Winslow also (*Exposition Anatomique de la Structure du Corps Humain*) directed his attention to them, for he observes: "Along the middle of each lateral surface of the small extremity is a tendinous or ligamentous band, three or four lines in width, and terminating at the pylorus"—*Tr. du Basventre*, §61. He, however, considers that they may lie externally to the muscular coat, in which view he is partly correct, as I shall presently endeavor to show.

Like the colon, the pyloric portion of the stomach is puckered; the ampullæ just now mentioned are formed by the shortness and strength of these longitudinal muscular fibres, resembling the pouches of the colon. As on the colon we very often see, at the sides of the longitudinal bands, the circular muscular fibres pass over the ampullæ in curves, which are closely pressed together in the two situations where the puckering takes place, but towards the bottoms of the ampullæ are further separated, according as the ampullæ are more distended.

In many cases we see these parts shining, like a smooth tendinous aponeurosis, which many authors also have remarked. I have sometimes examined this shining part, and found it composed, as Winslow mentioned, of a thin tendinous tissue in this peritoneal membrane, which is here abundantly provided with fibres of elastic tissue. This tendon-like formation, which in man is so imperfectly developed, and not unfrequently is wanting, acquires a greater importance from the fact that it is found strongly developed in many animals.

By far the thickest portion of the muscular coat is that of the extremity of the stomach bordering on the pylorus; the longitudinal fibres here again form a dense layer, evenly investing the entire part, as on the lower portion of the rectum. This small part of the stomach nearest to the pylorus constitutes, as it were, a little division in itself, and, according to my experience, is that which is least likely to be absent.

In the long form, this division of the stomach looks like an intestine, and is sometimes mistaken for a part of the duodenum; in many stomachs, which have been sent to me for examination, it has even been cut away. It occurs most frequently in women. It has, for the most part, only one ampulla on the lesser arch, but, on the contrary, two on the greater, of which the posterior is the great flexure, separated by a more distinct indentation from the remainder of the stomach.

In the third, or conical form, the great flexure usually appears as if removed nearer to the pylorus, and the greater ampulla in the lesser arch is small. The two other ampullæ, situated next the pylorus, are small, especially that in the lesser arch; and the little division just mentioned, situated next the pylorus, is better marked than in the two preceding forms.

In the new-born child, whose stomach is more rounded, I have not seen these ampullæ or indentations. Still, even in it, the part of the antrum next the pylorus is separately developed into a short cylindrical tube of about one centimetre in length, with thick walls, the thickness of which depends chiefly on a powerful circular, muscular belt. The valve of the pylorus is less developed than is ordinarily the case in adults; the muscular coat is thickest on the side belonging to the greater arch.

In a great number of the mammalia this part of the stomach forms, as has been already mentioned, a still more decided division than in man, as will be seen by glancing at the representation given even in Cuvier's *Lectures on Comparative Anatomy*, and in Meckel's translation, illustrated by a number of figures. Of the animals which are nearest to our hand, the dog and the cat tribe exhibit the part in question as a long, slender, bent cone; the horse, as a peculiar, rounded, thick part; in the pig it exhibits two constant ampullæ; in the hare it has, with the ampullæ, a small, reddish, thick, very muscular part next the pylorus; in the porpoise it constitutes a proper stomach, which is by Cuvier called the third stomach. In birds it forms the remarkable muscular stomach, which occurs likewise in many lizards; traces of the same are also found in various serpents.

As to the tendon-like parts, which are comprised in the above-named "ligamenta pylori," (Helvetius and Winslow,) they occur considerably developed in a number of mammalia, as the dog, the bear, the hare, &c.; in a great proportion of birds, especially those which live on seeds and insects, this tendinous formation is so well marked that it must be generally known; as it must be well known that this tendinous formation is found in the muscular stomach of crocodiles and several amphibia. Among fishes I have found it in the siluridæ. From all this it may be inferred that the tendinous formation spoken of deserves special attention, and must, in many cases, play an important part.

In a previous communication I have shown that cases occur in the human subject in which the valve of the pylorus almost disappears. I have, since that communication was written, found that this valve is constantly absent in a great number of mammalia and other vertebrate animals. On the other hand, there is in general found a broad, thick, circular, muscular formation around the greater part of the antrum pylori, which probably keeps the stomach closed throughout some extent, like the action of the circular muscles in the œsophagus and rectum.

As I have already mentioned, the duodenum likewise has a peculiar cavity, which probably has a special function. I have thought that this part ought to have a particular name, and have called it *antrum*, or *atrium duodeni*. The commencement of this part of the intestine is, in fact, as well in man as in a great number of mammalia, often separately rounded, wants the valvulæ conniventes on its inner surface, and has small villi, and large Brunnerian and Lieberkuhnian glands. In the porpoise this cavity is, as has just been mentioned, so distinct that it has been regarded as constituting a division of the stomach.—*Dub. Quart. Journ. of Med. Sci.* Aug. 1856, from *Hygiea*, Dec. 1855.

